# Metamaterial-Embedded Low SAR PIFA for Cellular Phone

**DOI:** 10.1371/journal.pone.0142663

**Published:** 2015-11-23

**Authors:** M. R. I. Faruque, M. I. Hossain, N. Misran, M. Singh, M. T. Islam

**Affiliations:** 1 Space Science Centre (ANGKASA), Universiti Kebangsaan Malaysia, 43600 UKM, Bangi, Selangor, Malaysia; 2 Department of Electrical, Electronic and Systems Engineering, Universiti Kebangsaan Malaysia, 43600 UKM, Bangi, Selangor, Malaysia; Northwestern Polytechnical University, CHINA

## Abstract

A metamaterial-embedded planar inverted-F antenna (PIFA) is proposed in this study for cellular phone applications. A dual-band PIFA is designed to operate both GSM 900 MHz and DCS 1800 MHz. The ground plane of a conventional PIFA is modified using a planar one-dimensional metamaterial array. The investigation is performed using the Finite Integration Technique (FIT) of CST Microwave Studio. The performance of the developed antenna was measured in an anechoic chamber. The specific absorption rate (SAR) values are calculated considering two different holding positions: cheek and tilt. The SAR values are measured using COMOSAR measurement system. Good agreement is observed between the simulated and measured data. The results indicate that the proposed metamaterial-embedded antenna produces significantly lower SAR in the human head compared to the conventional PIFA. Moreover, the modified antenna substrate leads to slight improvement of the antenna performances.

## Introduction

Currently, cellular phones represent an indispensable part of modern life. The most essential component of a cell phone is the antenna, which transmits and receives electromagnetic waves. Recently, the biological effects of electromagnetic (EM) energy radiated from cell phone antennas have attracted interest in the research community, because such devices are used in close proximity to the human head [1]. Either short term effects or long term effects may be caused by exposure to EM energy during cell phone use. To date, the World Health Organization (WHO) ruled out the possibility that radiation from a mobile phone could cause cancer in the user [2], [3]. The EM absorption in the human head from the radiated antenna on a mobile phone is measured in terms of the SAR, which is defined as the power absorbed by a unit mass of body tissue [4]. The mass measured for SAR evaluation corresponds to a mass of 1 g or a mass of 10 g of body tissue. The authoritative body that regulates the dose metric of the safe SAR absorbed in a human is the International Commission on Non-Ionizing Radiation Protection (ICNIRP). This regulatory body issued the standard SAR limit of 2 W/kg over 10 g of body tissue [5]. Another authoritative body, the Federal Communications Commission (FCC), determine the SAR safe limit of 1.6 W/kg over 1 g of body tissue [6]. Therefore, many research groups are going to bring down the SAR of the various portable devices.

The SAR values may vary due to the variation in EM source geometry, frequency of operation, properties of the body tissues, and the medium between source and human body [7]. The SAR values also depend on the antenna geometry and the radiated characteristics [8]. Moreover, the handsets with built-in antennas (internal antenna) have different SAR values than those of handsets with external antennas [9]. Various techniques have been discussed in the last decade to reduce of the Specific Absorption Rate (SAR) of cellular phone. Those methods can be separated into two broad categories: low SAR antennas and antennas with the auxiliary antenna element [10]. In [11], a novel structure of PIFA was proposed with an additional thin metal shim-layer in between the patch and the chassis with conducting sidewalls attached at the free-sides of the antenna. The proposed modified antenna structure could bring down the SAR significantly, only the antenna bandwidth was cut dramatically. The additional layer also detuned the resonant frequencies greatly. In [12], a two patch EBG structure was used as an additional antenna element. The results indicated the reduction in the SAR values toward the human head in the personal communications service (PCS) frequency band. Recently, metamaterials have been used to reduce the SAR of mobile phone antennas because metamaterials process exotic electromagnetic properties that are not normally found in nature. Metamaterials are artificially constructed materials that exhibit negative permittivity, negative permeability, or both simultaneously in a certain frequency range. In [13], the metamaterial based on a square split ring resonator (SSRR) was proposed as an additional antenna element for SAR reduction. This investigation achieved the SAR reduction in the GSM 900 MHz band. In [14], a metamaterial-based handset PIFA antenna in the form of a low SAR antenna in the WiMAX (2.4 GHz) band was developed by adding an extra meta-surface in the ground plane of the antenna. In [15], a modified metamaterial-inspired low SAR antenna was proposed; in this antenna, a z-element was added above the patch of the PIFA antenna. These additional antenna elements can reduce the SAR, but it surely increases the antenna volume. In [16], a triangular-shaped split ring resonator (SRR) surface was used in between the PIFA and the human head to reduce the SAR at GSM 900 MHz.

In this paper, a new approach of a metamaterial-embedded PIFA antenna is introduced to reduce the SAR in the human head. In this approach, the ground plane of the PIFA is modified using a metamaterial array and the SAR values of the proposed antenna are compared with those of the conventional PIFA antenna. The metamaterial is embedded in the antenna in such a way that the antenna volume is not affected; this approach can be easily implemented in modern cellular phones.

## Models and Techniques

### Metamaterial antenna

**Fig 1:**
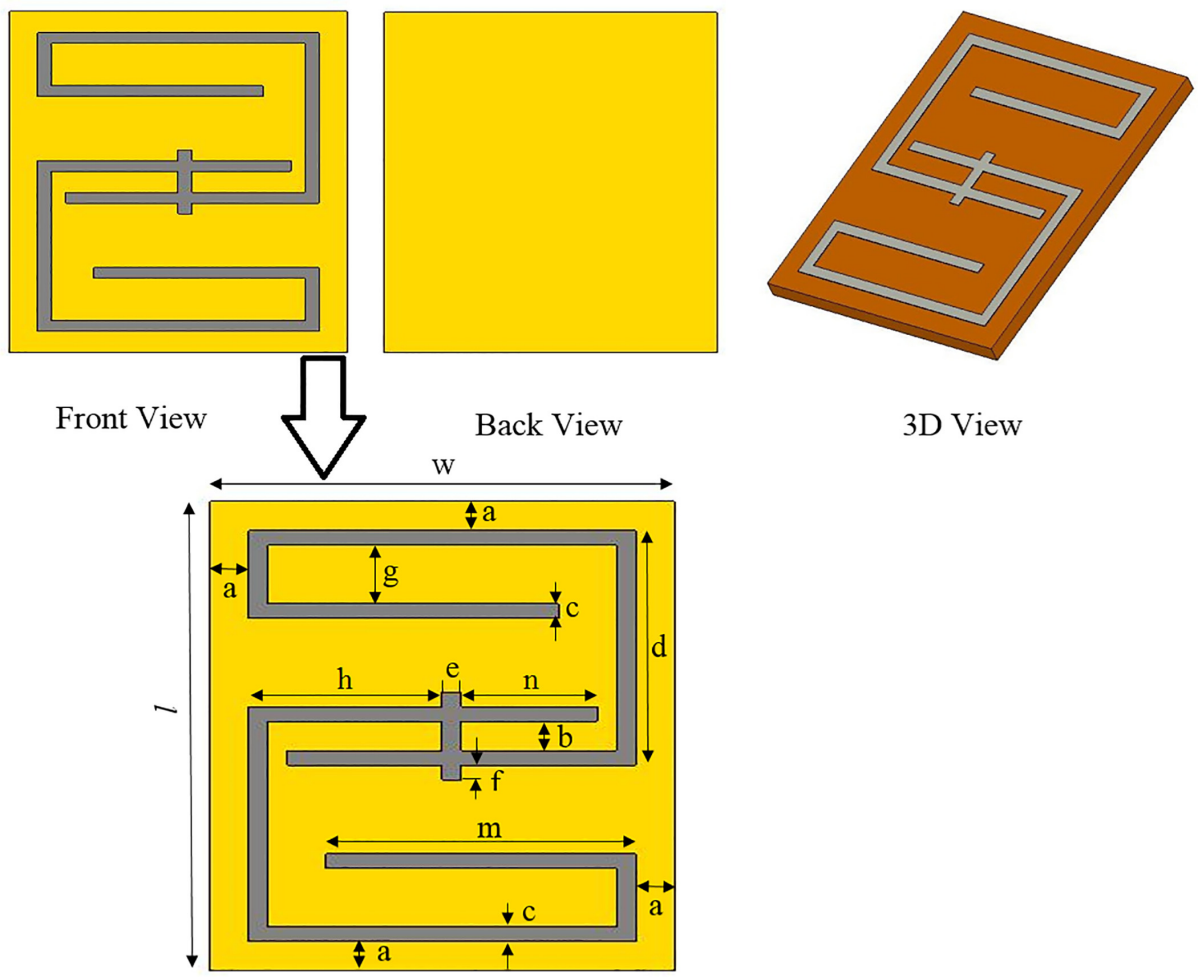
The geometry of the metamaterial unit-cell.

**Fig 2:**
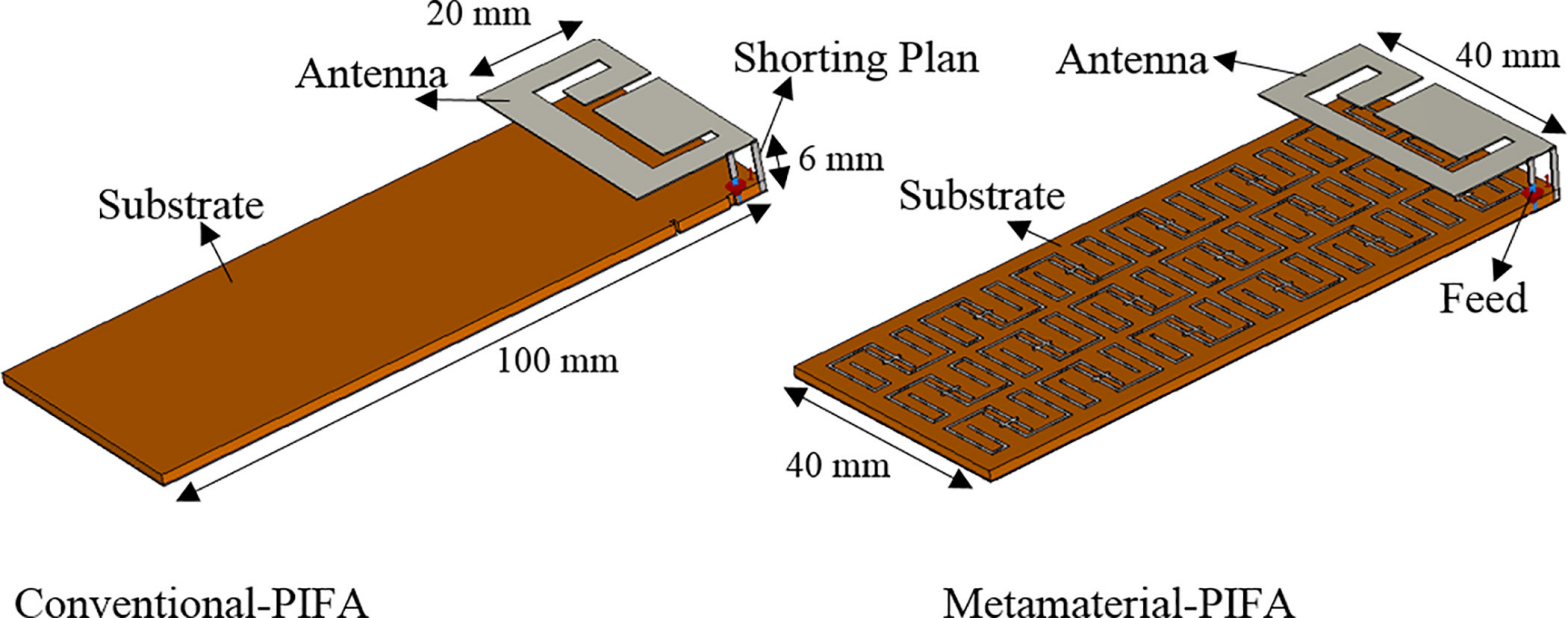
The geometry of the conventional (left) and the metamaterial-embedded (right) PIFA.

**Fig 3:**
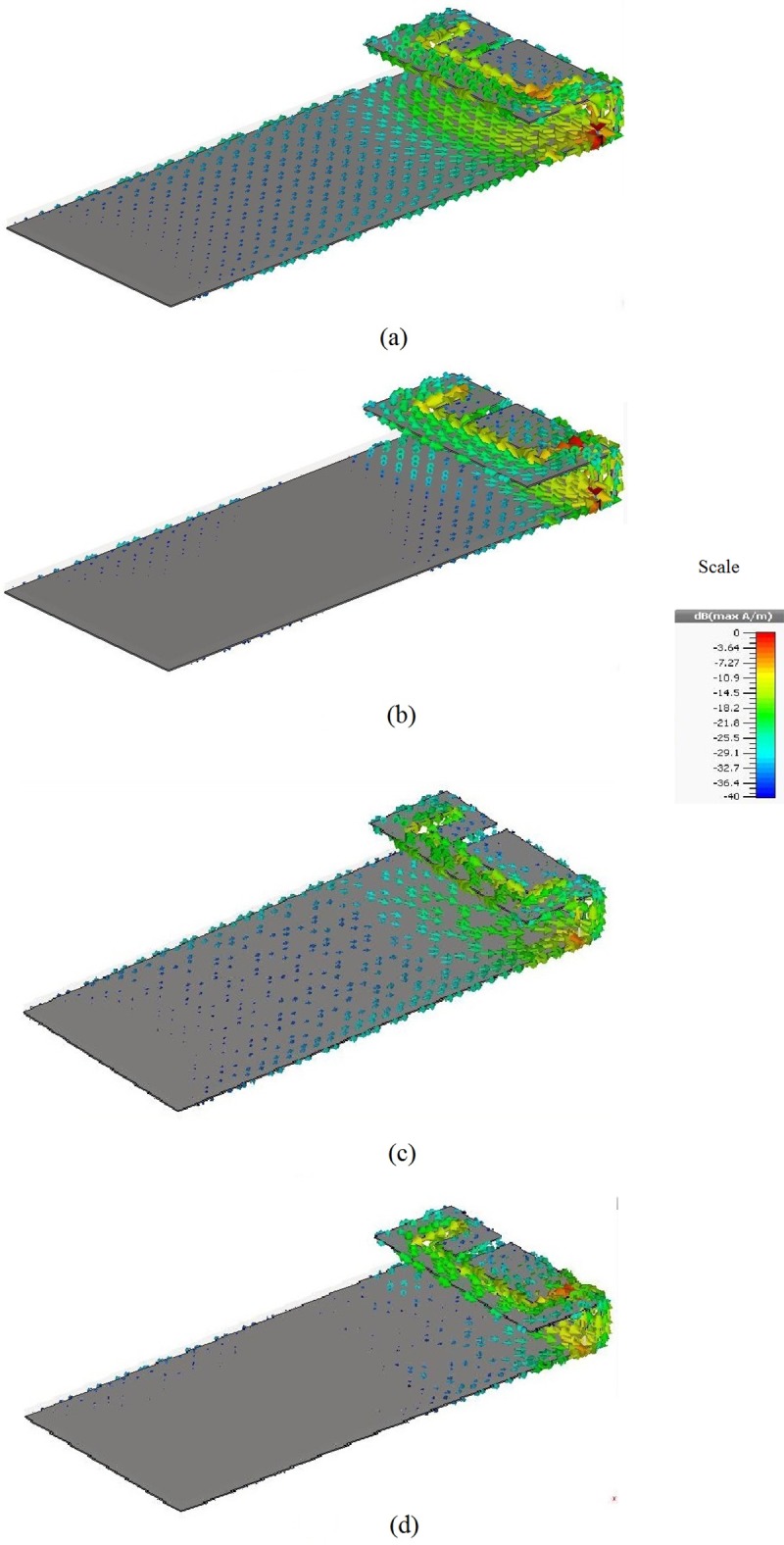
Surface Current distribution: (a) conventional PIFA at 0.9 GHz, (b) conventional PIFA at 1.8 GHz, (c) Metamaterial-embedded PIFA at 0.9 GHz, and (d) metamaterial-embedded PIFA at 1.8 GHz.

Fig 1 shows the structures of the metamaterial unit-cell. The metamaterial unit-cell geometry is based on [17]. The dimensions of the metamaterial unit-cell are 16 mm × 12 mm × 0.8 mm while the original unit-cell dimension was 12 mm × 12 mm × 0.8 mm. On the other hand, the connecter in the central of structure is modified from the original design. Because, the original design of metamaterial unit-cell was not compatible with cellular phone frequencies. Hence, the specific parameters are readjusted for better performance using optimization processes. The design specification of metamaterial is listed in Table 1. The one layer planar metamaterial array dimensions are 96 mm × 36 mm × 0.8 mm, and it is designed as an arrangement of 3 × 6 unit-cells. The metamaterial array is fabricated on a 0.8 mm thick FR-4 substrate using printed copper strips. The conventional and metamaterial PIFA geometries are shown in Fig 2. The PIFA is designed to operate at the dual bands of GSM 900 MHz and DCS 1800 MHz. The conventional PIFA consists of an E-shaped patch with dimensions 40 mm × 20 mm × 0.3 mm, one shorting wall, and the substrate. The separation between the patch and the ground plane is 6 mm. The 0.8 mm thick sheet of FR-4 material is used as the antenna substrate. The antenna patch, the shorting wall and the feed are made of copper sheets (0.3 mm thickness). The conventional PIFA substrate is replaced by the metamaterial array to construct the metamaterial PIFA. Fig 3 indicates the surface current distribution of proposed antemnna. It is observed that the ground plane length highly affects a lower frequency band of proposed antenna. On the other hand, the metamaterial-embedded ground plane alters the current distribution slightly for both upper and lower frequency bands compared to the without a metamaterial ground plane.

**Table 1:** Design specifications of metamaterial unit-cell.

Parameters	Value (mm)	Parameters	Value (mm)
a	1	g	2
b	1	h	5
c	0.5	*l*	16
d	8	m	8
e	0.5	n	3.5
f	0.5	w	12

### Human head and phone model

**Fig 4:**
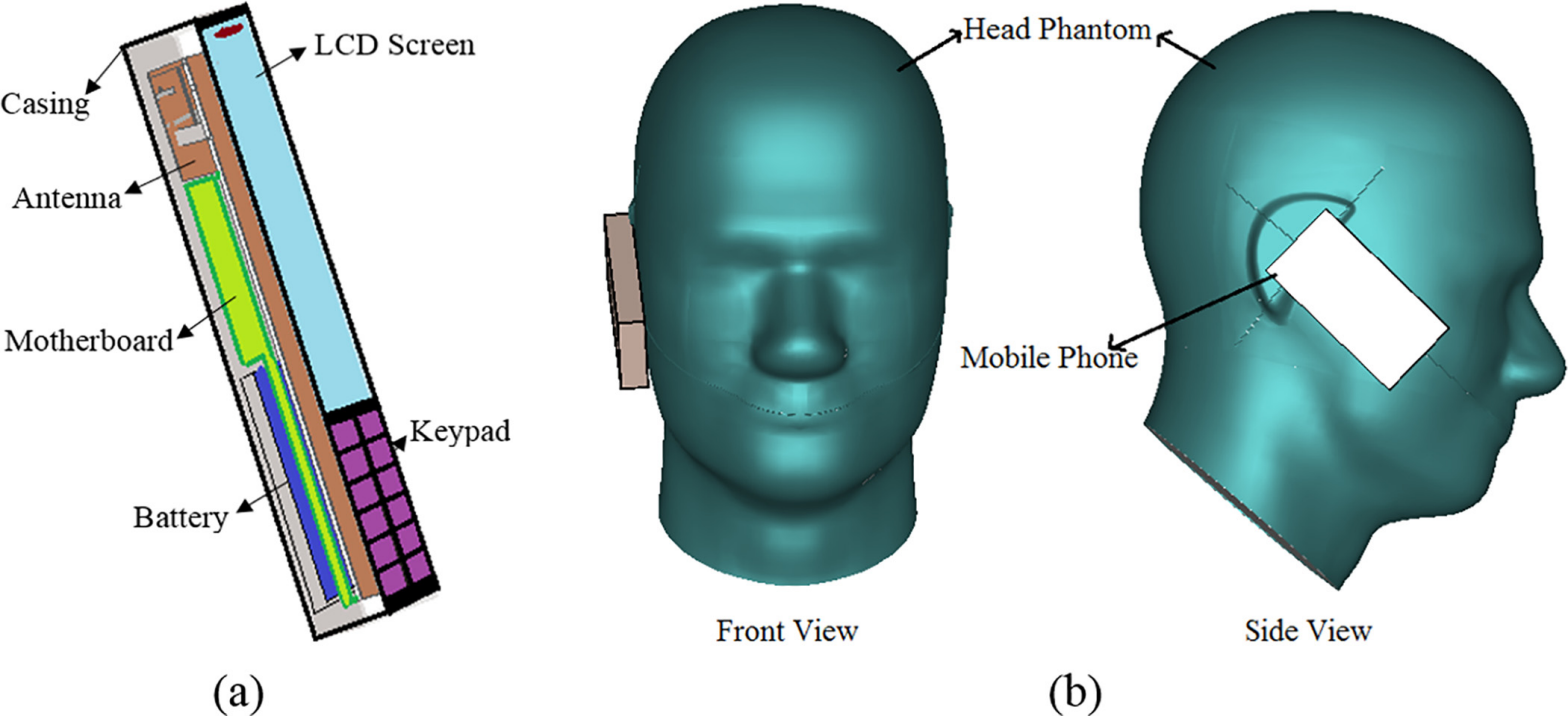
(a) Mobile phone model with antenna and (b) SAM phantom with mobile phone.

In this study, a numerical Anthropomorphic Mannequin (SAM) head model [18] is employed with a mobile phone to assess the interaction between the EM radiation and the human head. The homogeneous head model was comprised of two layers, the inner and outer layers, with specific dielectric properties at a particular frequency exposure. For exposure to different frequencies, the head exhibited different dielectric properties. These properties were constant as long as the head was exposed to the same frequency exposure. Table 2 lists the properties of SAM head at 900 MHz and 1800 MHz. A plastic box is used as a cover of the mobile phone antenna in the cheek and tilt holding positions with the head phantom [19]. The thickness of the plastic sheet is 0.25 mm, and the dimensions of the case box are 102 mm × 42 mm × 12 mm. Fig 4(A) and 4(B) show the mobile phone model with antenna and SAM head model, respectively. The dispersive models were used to simulate the properties of all the dielectric materials, which enables the characterization of the performance over a broad frequency band.

**Table 2:** The material properties used in the simulation.

SAM Material	900 MHz		1800 MHz	
	ε_r_	σ (S/m)	ε_r_	σ (S/m)
SAM shell	3.7	0	3.5	0.0016
SAM liquid	41.5	0.97	40	1.42

### Methods

This investigation was performed using a finite integration technique (FIT), which is performed using the commercial software from computer simulation technology (CST) microwave studio (MWS) [20]. To obtain the scattering parameters of the metamaterial, both perfect electric and magnetic boundaries are imposed on the unit cell and the tested unit cell was placed between two waveguide ports. The perfect electric conducting and perfect magnetic conducting boundary conditions are defined in the x- and y-directions and the structure are excited by a uniform plane wave propagating toward the z-direction. The Nicolson Rose Weir (NRW) method [21], [22] is used for the extraction of the effective relative permittivity (*ε*_*r*_), permeability (*μ*_*r*_) and refractive index (*n*_*r*_) from the scattering parameters (S_11_ and S_21_). Formulas (1), (2), and (3) are used to calculate the effective permittivity, the permeability and the refractive index respectively [17].

$$\mu_{\text{r}}=\frac{2c(1-S_{21}+S_{11})}{j\omega d(1+S_{21}-S_{11})}$$ (1)

$$\varepsilon_{\text{r}}=\mu_{\text{r}}+j\frac{2cS_{11}}{\omega d}$$ (2)

$$n_{\text{r}}=\sqrt{\mu_{\text{r}}\varepsilon_{\text{r}}}$$ (3)

where c is the velocity of light, d is the thickness of substrate, and ω = 2π*f* is the angular frequency.

The SAR values are calculated in the post-processing phase of the simulation using the IEEE standard algorithm. The SAR calculated via the simulation was determined using the following equation [19]:

$$SAR=\frac{\sigma E^{2}}{2\rho }$$ (4)

where *E*, *σ*, and *ρ* denote the induced electric field strength, conductivity of tissue, and density of the tissue.

## Results and Discussions

In this investigation, the metamaterial-embedded PIFA is proposed to make a low SAR antenna. The comparison between the conventional and the metamaterial PIFA considers the antenna performance and the SAR characteristics. The results are presented in following three categories.

### Effective parameters of metamaterial

**Fig 5:**
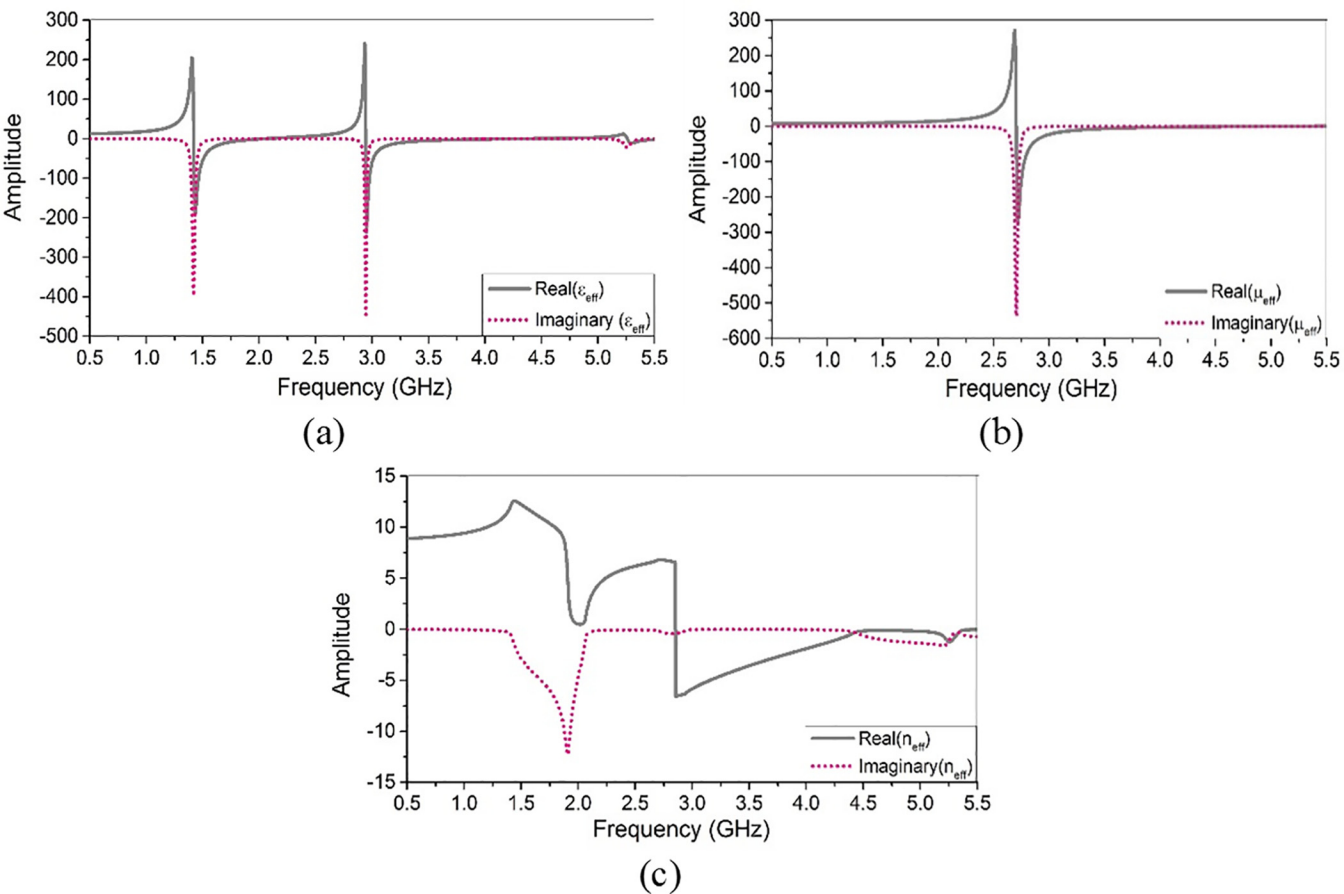
Effective parameters of the metamaterial: (a) effective permittivity, (b) effective permeability, and (c) effective refractive index.

The effective permittivity, the effective permeability, and the effective refractive index of the metamaterial unit-cell are shown in Fig 5(A), 5(B) and 5(C), respectively. All effective parameters are plotted comprising both real and imaginary parts. According to the real parts of effective parameters, the metamaterial unit-cell exhibits double negative characteristics (both $\varepsilon_{r}^{eff}$ and $\mu_{r}^{eff}$ negative) from 2.9 to 5 GHz. The effective refractive index (*n*_*eff*_) of unit-cell exhibits a negative value from 2.8 to 5.5 GHz. The designed metamaterial unit-cell also exhibits a single negative value ($\varepsilon_{r}^{eff}$ negative) from 1.4 to 2.2 GHz. The metamaterial array exhibits similar results with slightly shifted curves of the effective permittivity and the effective refractive index.

### Antenna performances

**Fig 6:**
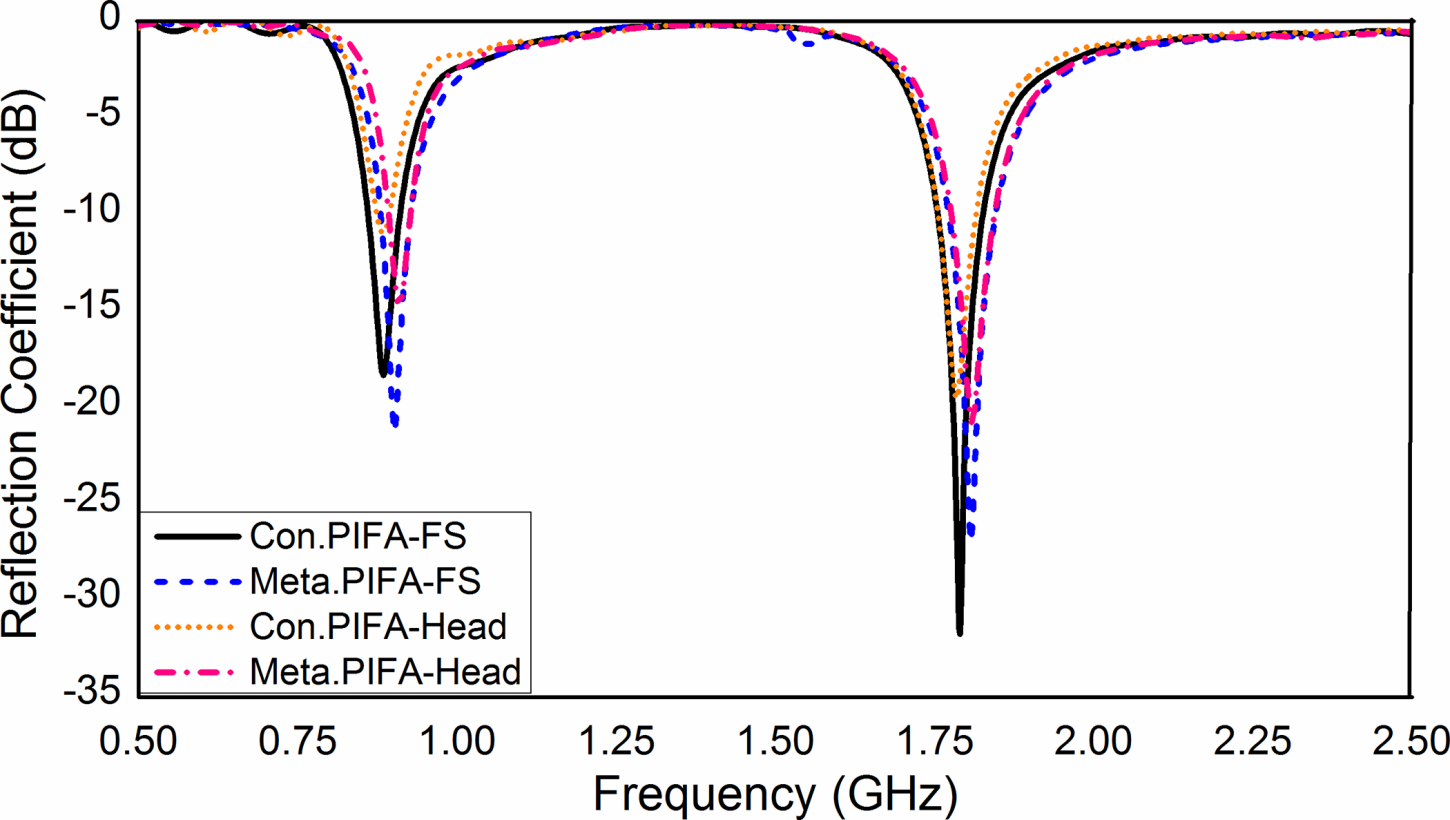
Reflection coefficient of antenna for different configurations.

The comparison of the antenna performances of conventional and metamaterial PIFA considers three different parameters: reflection coefficient (S_11_), radiation efficiency and gain. All those performance parameters are recorded considering the antenna in free-space and the antenna with head phantom for both antennas. Fig 6 shows the curve of the reflection parameters (S_11_) for both the conventional and the metamaterial PIFA. The results show that the metamaterial-embedded substrate does not affect the antenna resonance points significantly. The patch of PIFA is located 6 mm away from the ground plane; hence, the metamaterial structure does not affect the antenna characteristics significantly. In addition, a 2 mm gap is kept between the metamaterial array structure edges and the edges of the ground plane. As a result, the shorting plane and the feed of the antenna is not affected by the metamaterial array. The S-parameter curve of the metamaterial PIFA with the head model is affected less than that of the conventional PIFA.

**Fig 7:**
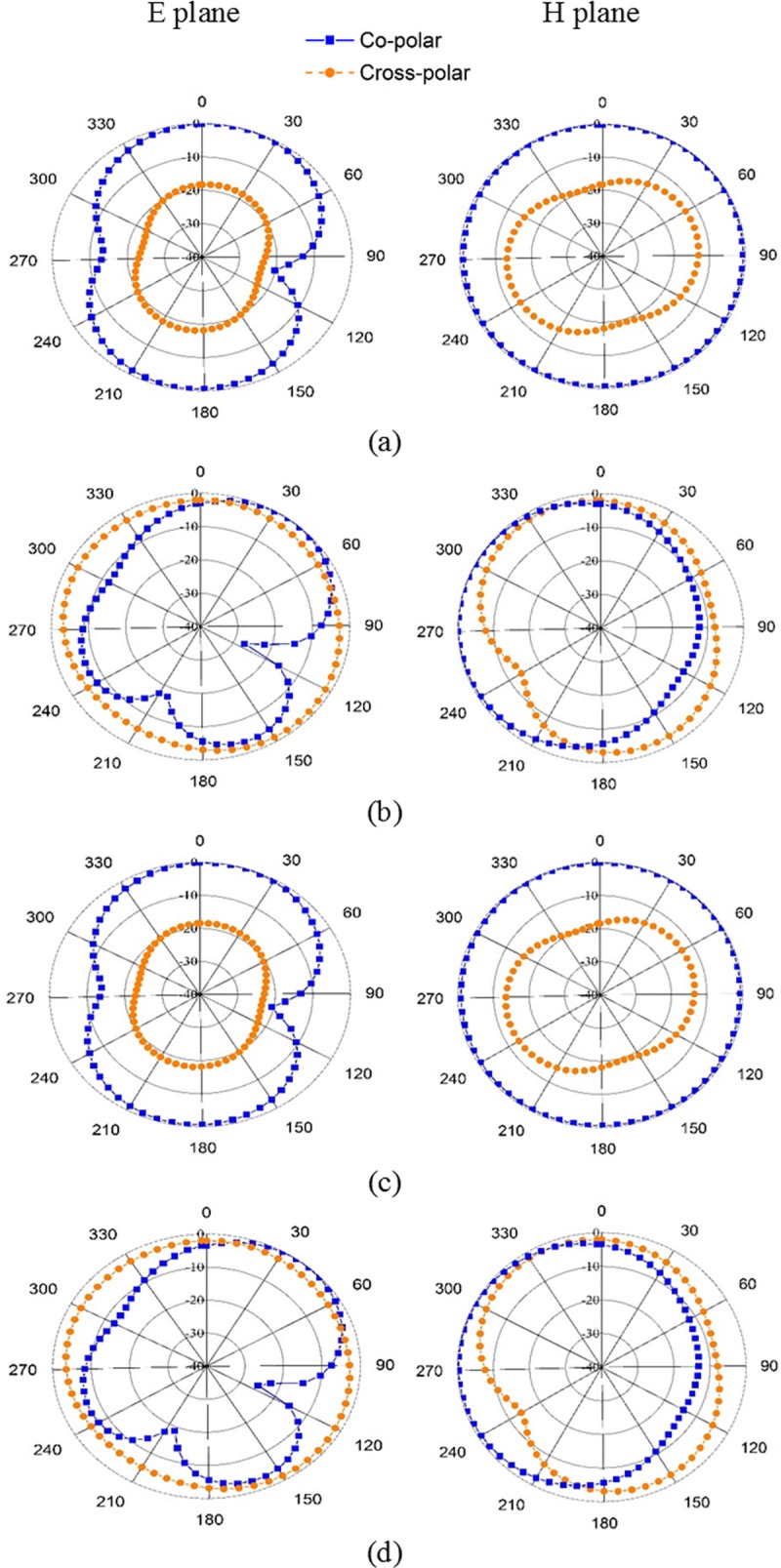
Gain radiation patterns for: (a) conventional PIFA at 0.9 GHz, (b) conventional PIFA at 1.8 GHz, (c) metamaterial PIFA at 0.9 GHz, and (d) metamaterial PIFA at 1.8 GHz.

The gain and radiation efficiency values are listed in Table 3 for both antennas for the two different configurations: antenna in free-space and antenna with head phantom. In the free-space configuration, the conventional PIFA exhibits 87.5% and 94.5% efficiency at 0.9 GHz and 1.8 GHz, respectively. The PIFA with the metamaterial ground plane shows higher radiation efficiencies than those of the conventional PIFA: the metamaterial PIFA shows 97.9% and 97.5% efficiency at 0.9 GHz and 1.8 GHz, respectively. For the antenna with the human head configuration, the radiation efficiencies are reduced for both antennas. The antenna gains for the conventional PIFA are 1.933 and 3.793 dB in free-space at the lower and upper frequency bands, respectively. Moreover, the metamaterial PIFA shows improvement in the gain: 2.31 and 3.83 dB at 0.9 and 1.8 GHz, respectively. The metamaterial PIFA also exhibits higher gain in the antenna with the head configuration for both frequency bands compared to the conventional PIFA. Due to the higher dielectric properties of the head phantom, the antenna impedance changes, thereby changing the antenna performance. Fig 7(A)–7 (D) shows the radiation pattern of the conventional and the metamaterial PIFA at 0.9 and 1.8 GHz. The results show the nearly omnidirectional pattern for both frequency bands. Results at other frequencies of both GSM bands exhibit very similar patterns as plotted, indicating stable radiation patterns are obtained. Moreover, the radiation pattern plots clearly indicate that the metamaterial ground plane does not alter the radiation pattern of the conventional PIFA.

**Table 3:** Radiation efficiency and gain.

Configuration	Radiation Efficiency (%)		Gain (dB)	
	0.9 GHz	1.8 GHz	0.9 GHz	1.8 GHz
Con.PIFA-FS	87.5	94.5	1.933	3.793
Meta.PIFA-FS	97.9	97.5	2.309	3.827
Con.PIFA-Head	33	66	0.309	5.13
Meta.PIFA-Head	37	70	0.56	5.504

### Specific absorption rate

The SAR analysis of conventional and metamaterial PIFA is performed using two holding positions of talk mode: cheek and tilt. In tilt position analysis, 15 degree tilt angle is considered. Table 4 exhibits 1 g and 10 g SAR values in the human head for both antennas. For the cheek position, conventional PIFA produces 3.021 W/Kg and 2.848 W/Kg 1g SAR at 0.9 GHz and 1.8 GHz respectively. For the metamaterial PIFA in cheek position, 3.015 W/Kg and 2.001 W/Kg 1g SAR are observed at 0.9GHz and 1.8 GHz respectively. It is found that the 1g SAR values for conventional and metamaterial PIFA are close to each other at 0.9 GHz for cheek position analysis. On the other hand, metamaterial PIFA exhibits a significant SAR reduction compared to conventional PIFA at 1.8 GHz. Moreover, the SAR values of the tilt position analysis show slightly lower values than that of cheek position analysis because of the fact that the effective distance from the antenna to human head is increased for the tilt position. In the tilt position analysis, metamaterial PIFA also exhibits nearly identical 1g SAR value at 0.9 GHz, but significant reduction at 1.8 GHz. The results indicate a similar tendency of the SAR values as 1g SAR. However, the metamaterial PIFA leads to reduce 42.33% and 66.85% 1g SAR and 10g SAR respectively at 1.8 GHz for cheek position. Similarly, 58.38% and 51.36% reduction in 1g SAR and 10g SAR are obtained due to the effect of metamaterial ground at 1.8 GHz for tilt position analysis. The proposed metamaterial PIFA exhibits the SAR reduction significantly at 1.8 GHz only due to the metamaterial embedded ground plane. Because, the presented metamaterial shows negative characteristics from 1.4 to 2.2 GHz and 2.9 to 5 GHz. Basically, negative permittivity of metamaterial leads to increase the surface impedance significantly and hence the surface current in the ground plane at 1.8 GHz is reduced. As a result, the induced electric field strength in the human head degrades. At the lower GSM (900 MHz) frequency band of the PIFA, proposed metamaterial is unable to affect the SAR values for the above reason.

**Table 4:** SAR values for conventional and metamaterial antenna.

Configuration	Position	0.9 GHz		1.8 GHz	
		SAR 1 g (W/kg)	SAR 10 g (W/kg)	SAR 1 g (W/kg)	SAR 10 g (W/kg)
Con.PIFA	Cheek	3.021	2.046	2.848	1.668
	Tilt	1.947	1.321	2.721	1.562
Meta.PIFA	Cheek	3.015	2.051	2.001	1.037
	Tilt	1.904	1.281	1.718	1.032

### Experimental Validation

**Fig 8:**
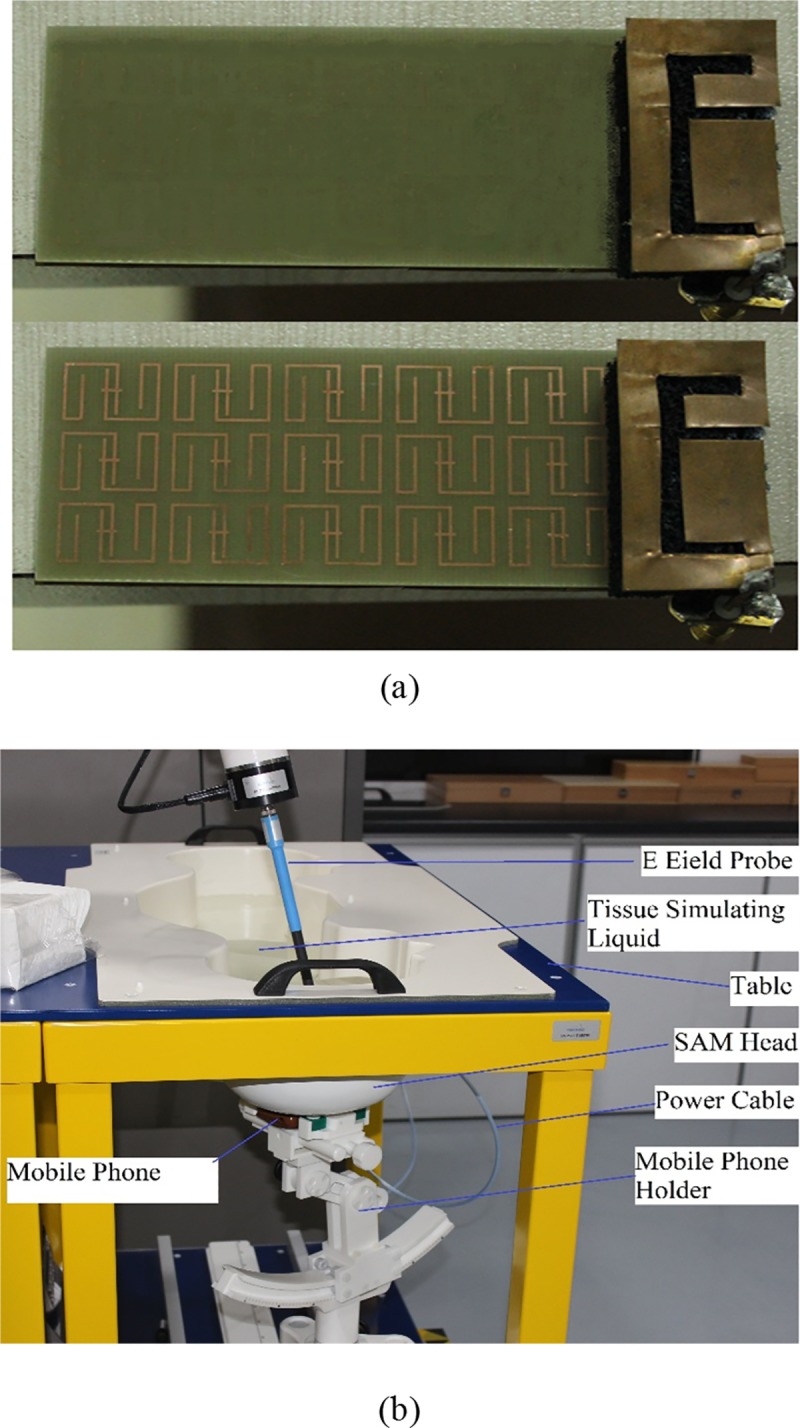
(a) Fabricated prototype of conventional (top) and metamaterial embedded (bottom) PIFA and (b) COMOSAR Measurement setup for SAR evaluation.

**Fig 9:**
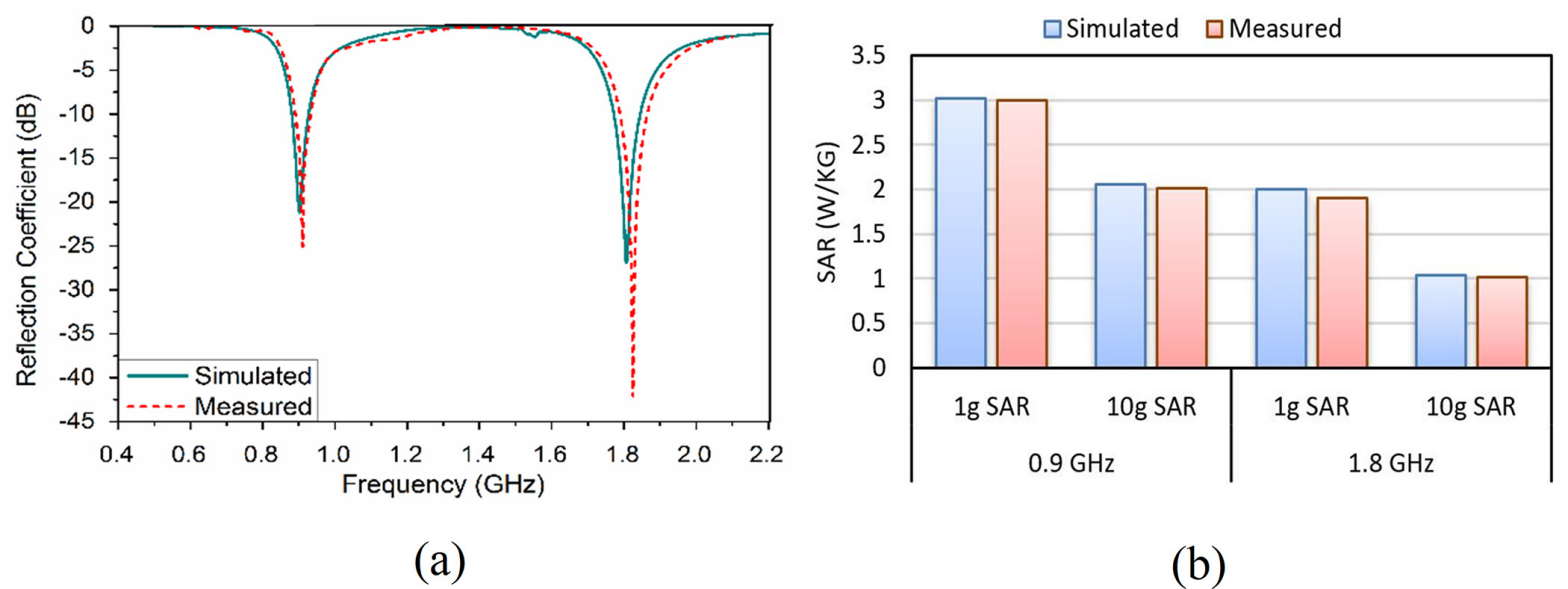
(a) Measured and simulated reflection coefficient of proposed metamaterial PIFA and (b) measured and simulated SAR values of proposed metamaterial PIFA.

In this section, the experimental results of SAR values and reflection coefficients are presented for both conventional and metamaterial PIFA. Fig 8(A) indicates the fabricated prototype of conventional and metamaterial PIFA. The reflection coefficient is measured in an anechoic chamber using Agilent N5227A Vector Network Analyzer (VNA). The SAR values are measured using the COMOSAR measurement system of SATIMO. The SAR measurement setup is indicated in Fig 8(B). The SAR measurement system consists of the following items: PC to control all the system, six-axis robot, data acquisition system, miniature E-field probe, mobile phone holder, flat SAM head phantom, and head simulating tissues. The system uses a robot to position the SAR probe (E-field probe) inside the head phantom. Fig 9(A) exhibits the comparison of measured and simulated reflection coefficient of proposed metamaterial PIFA. The results indicate very good agreement between measured and simulated data. A little bit shift of resonant frequency is observed in case of measured reflection coefficient, which is occurred mainly due to the fabrication error and connector issue. Fig 9(B) indicates the comparison of measured and simulated SAR values in the human head in the case of proposed metamaterial PIFA. Only the measured SAR value considering cheek position is presented in this paper. The results show very good agreement between measured and simulated SAR in the human head.

## Conclusion

In this paper, a new approach of metamaterial PIFA is proposed to reduce the peak SAR in the human head. The substrate of convention PIFA is modified using a DNG metamaterial array to make new metamaterial embedded PIFA. The SAR analysis is performed using two different holding positions of talk mode: cheek and tilt. The results indicate that the metamaterial ground plane improves the radiation efficiencies and gains compared to conventional PIFA. Moreover, the SAR results indicate significant reduction in the SAR values at the upper frequency band for both cheek and tilt position of talk mode. Furthermore, future studies may be extended to develop metamaterial, which will be perfectly compatible with mobile phone frequency bands.
